# Supplementary figures and images for: Physical activity prevents acute inflammation in a gout model by downregulation of TLR2 on circulating neutrophils as well as inhibition of serum CXCL1 and is associated with decreased pain and inflammation in gout patients
Source: PLoS One. 2020 Oct 1;15(10):e0237520. doi: 10.1371/journal.pone.0237520 (PMC7529261; doi:10.1371/journal.pone.0237520)

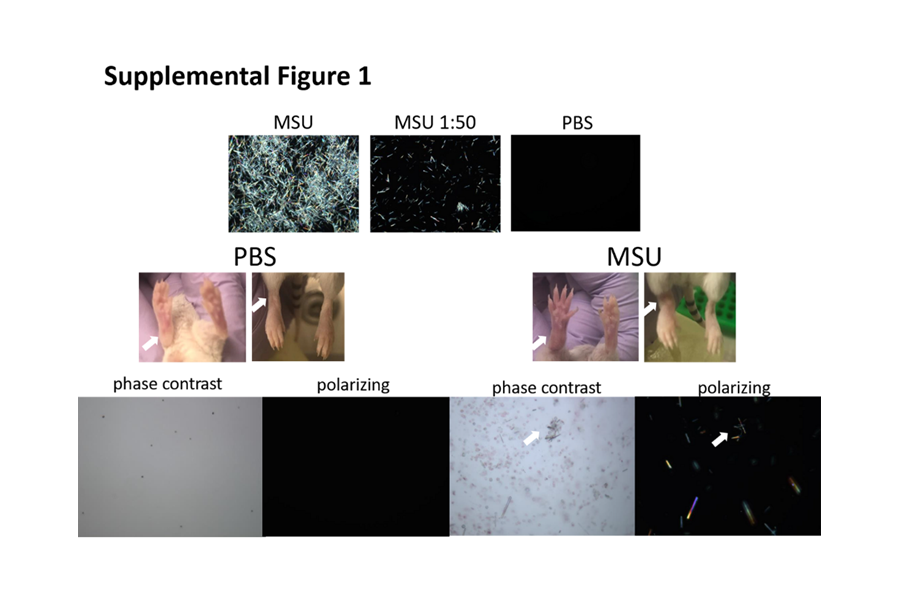

Supplement: S1 Fig — Confirmation of MSU crystals in the ankle joints of MSU injected mice (top row). MSU crystals were visualized by polarizing light and phase contrast microscopy following resuspension in PBS, diluted 1:50, or PBS without MSU. Mice were given intra-articular injections of PBS or MSU and assessed 16 hrs later. (middle row) Pictures taken of feet/ankles show increased swelling with MSU injection relative to contralateral feet/ankles or PBS controls (white arrows). (bottom row) Synovial aspirates of PBS-injected mice show no detectable cells or MSU crystals. Conversely, MSU injection produced detectable MSU crystals and cells were observed actively engulfing crystals (white arrow). (TIF) [file pone.0237520.s001.tif]

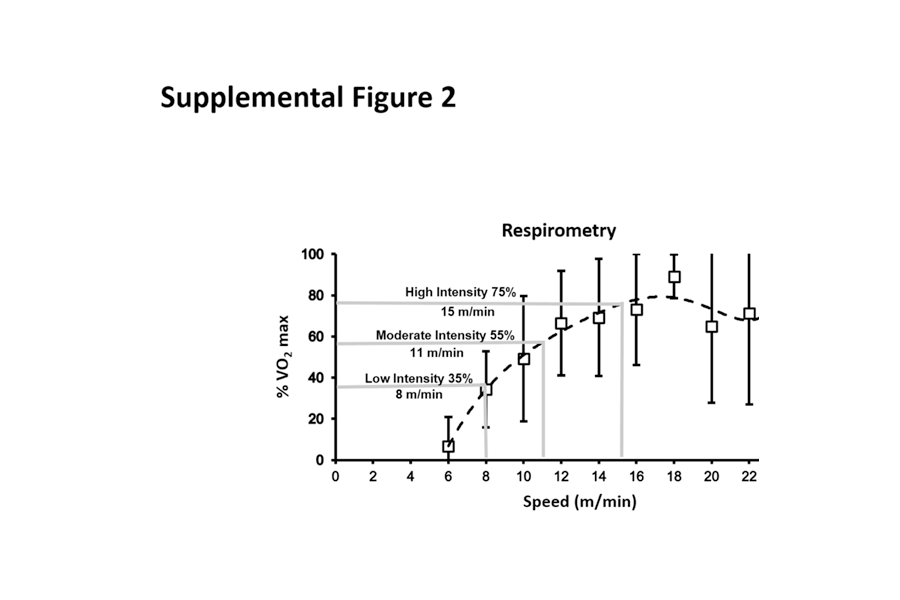

Supplement: S2 Fig — Male and female Balb/c mice (n = 14) were exercised in five minutes intervals at increasing speeds with a 5% incline. The VO2 consumption was recorded every 30 seconds. Values were plotted and a polynomial curve was applied to graph and interpolate speeds correlating with 35% (low-intensity), 55% (medium-intensity), and 75% (high-intensity) VO2 max. Speeds were determined to be 8 m/min, 11 m/min, and 15 m/min, respectively. (TIF) [file pone.0237520.s002.tif]

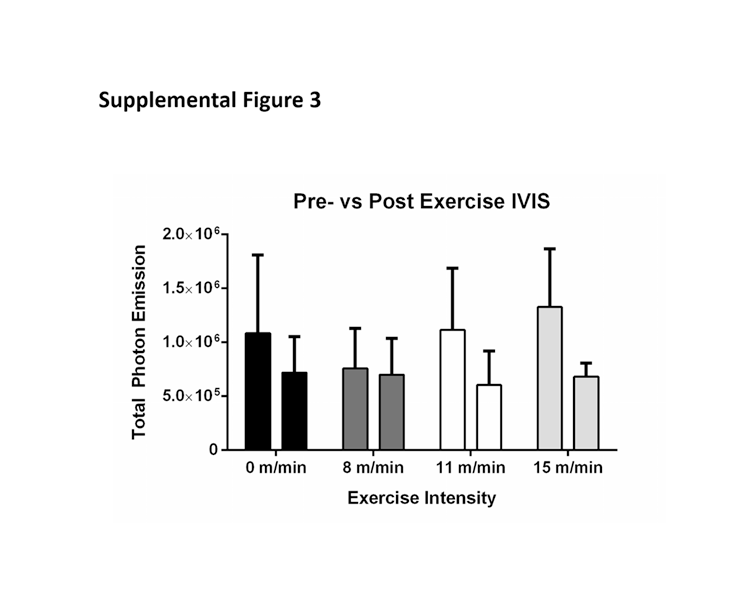

Supplement: S3 Fig — Male and female Balb/c mice (n = 14) were exercised at 8 m/min, 11 m/min, and 15 m/min daily for 2 weeks. Systemic NF-κB activity was measured by IVIS before beginning the exercise regimen and 24 hrs after the completion of the final session. Analysis by ANOVA and followed by two-tailed, nonparametric, unpaired Mann-Whitney t-tests produced no statistically different results. (TIF) [file pone.0237520.s003.tif]

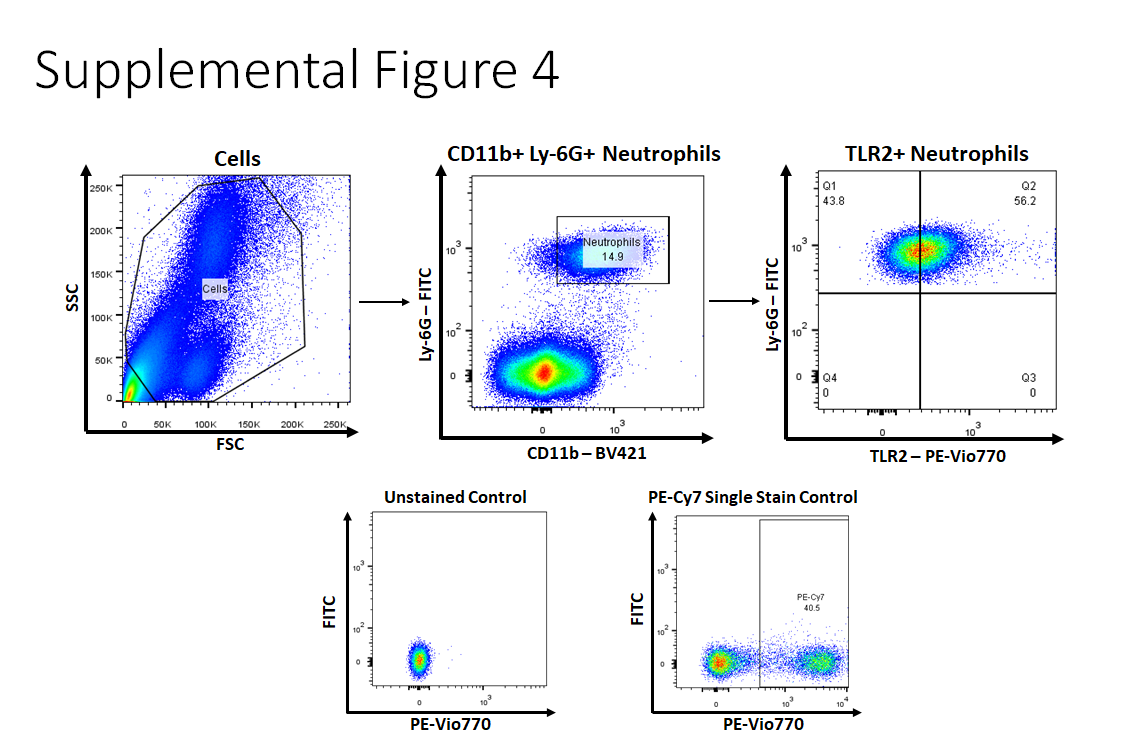

Supplement: S4 Fig — Gating strategy of successive flow cytometry gates to detect TLR2 positive expression. The total cell population was first gated on forward and side scatter. Subsequently, neutrophils were gated by Ly6G+ / CD11b+ detection. TLR2 expression was then determined by PE-Vio770 fluorochrome detection relative to the signals observed in unstained and single stained cells. (TIF) [file pone.0237520.s004.tif]

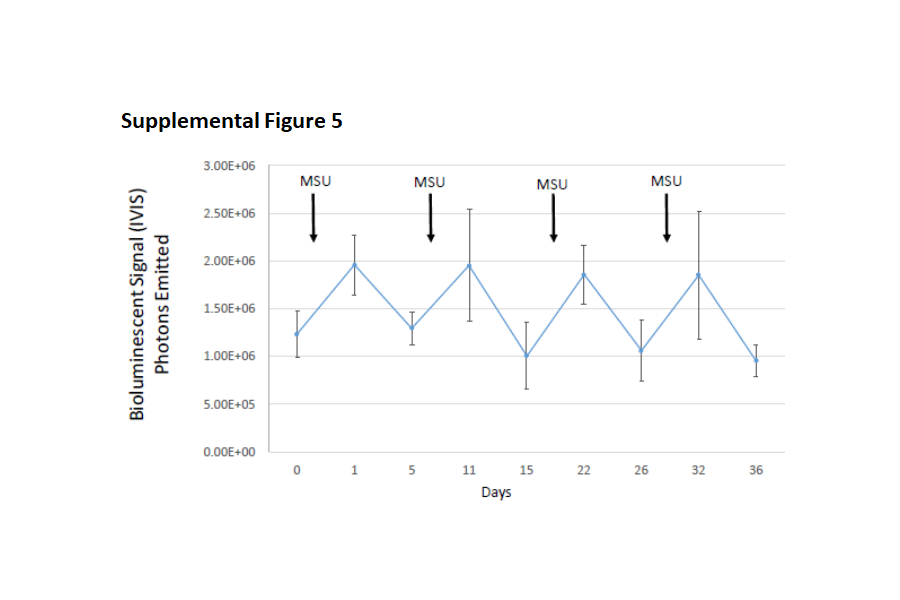

Supplement: S5 Fig — Bioluminescent imaging of NF-κB-RE-luc mouse feet measured and quantitated via IVIS. Repeated intra-articular injections of MSU crystals (20 μg) were made where indicated. Analysis of localized NF-κB activity suggests complete resolution prior to subsequent challenges. (TIF) [file pone.0237520.s005.tif]

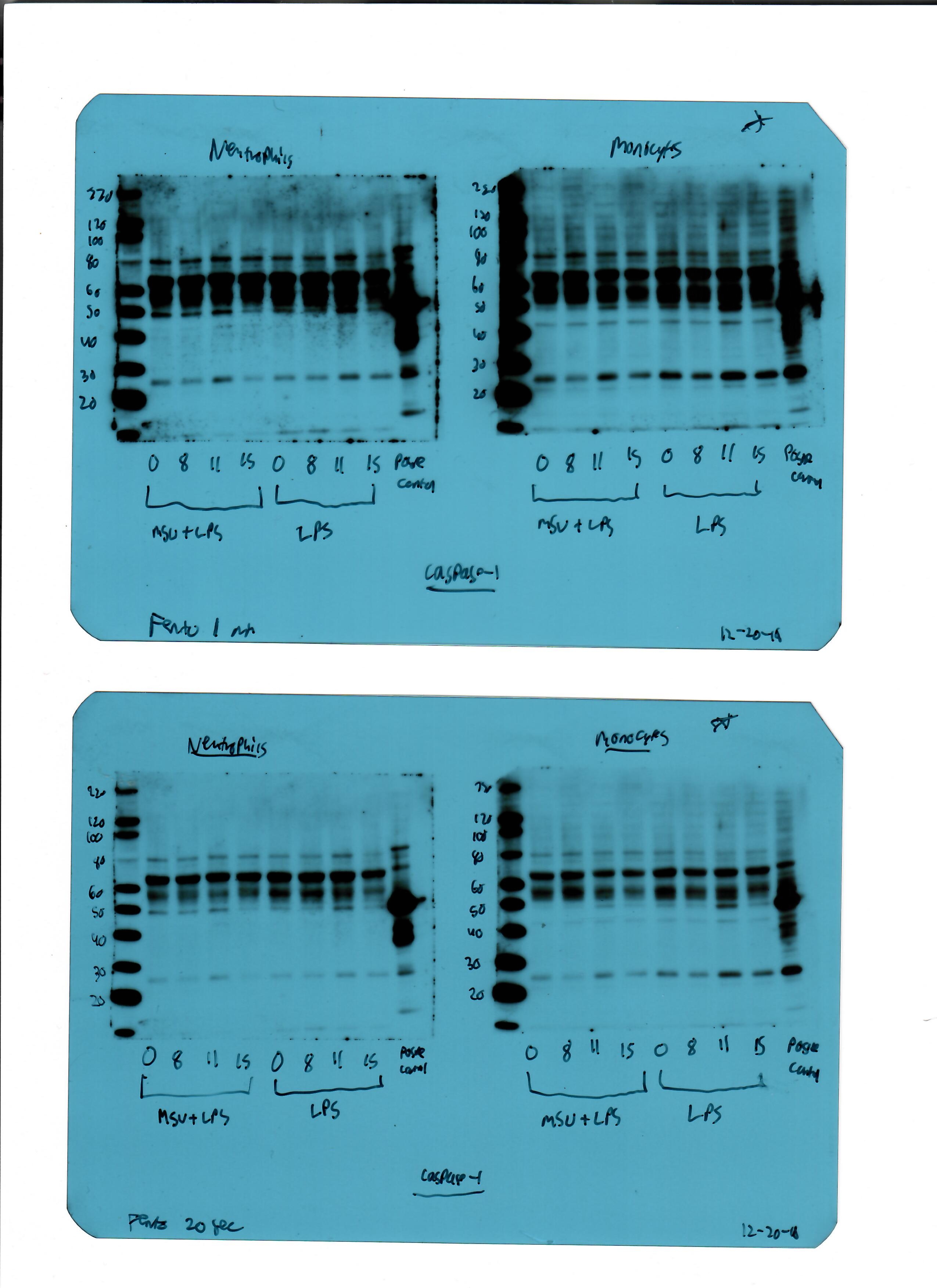

Supplement: S1 Raw data — (ZIP) [file pone.0237520.s006.zip › raw data files/12-20-18 Caspase-1__western blot.jpg]

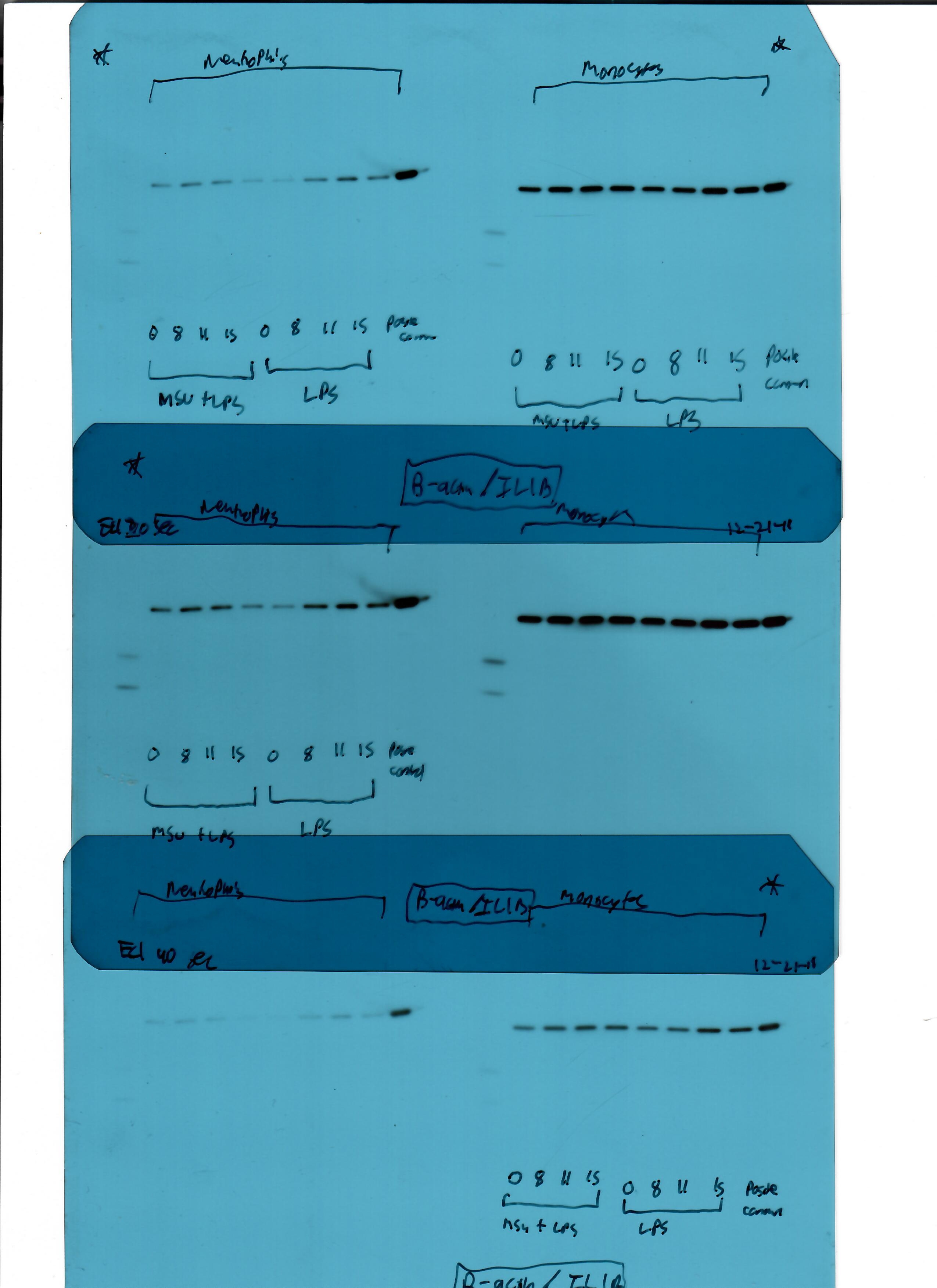

Supplement: S1 Raw data — (ZIP) [file pone.0237520.s006.zip › raw data files/12-21-18 Beta actin__western blot.jpg]

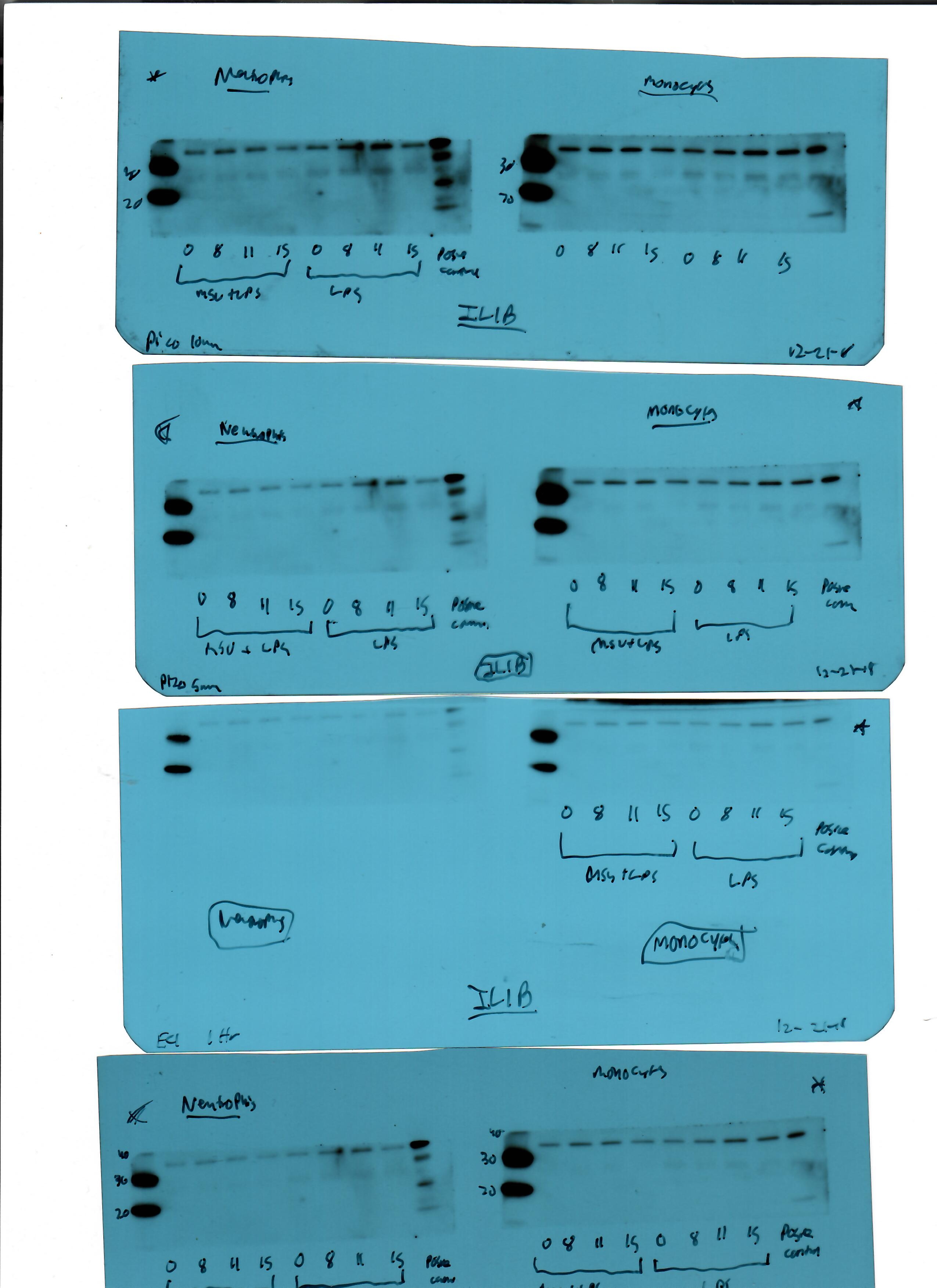

Supplement: S1 Raw data — (ZIP) [file pone.0237520.s006.zip › raw data files/12-21-18 IL1b__western blot.jpg]
