# Supplementary material for: Physical activity prevents acute inflammation in a gout model by downregulation of TLR2 on circulating neutrophils as well as inhibition of serum CXCL1 and is associated with decreased pain and inflammation in gout patients
Source: PLoS One. 2020 Oct 1;15(10):e0237520. doi: 10.1371/journal.pone.0237520 (PMC7529261; doi:10.1371/journal.pone.0237520)
Supplement: S1 File — (DOC) [file pone.0237520.s007.doc]

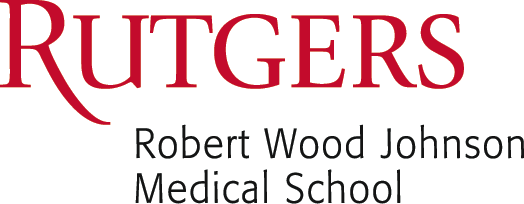


Division of Rheumatology

Robert Wood Johnson Medical School

Rutgers, The State University of New Jersey

One Robert Wood Johnson Place—MEB 484

New Brunswick, NJ 08903

p. 732-235-8378

f. 732-235-7238

**SUBJECT CONSENT TO TAKE PART IN A RESEARCH STUDY**

**TITLE OF STUDY:** Exercise in gout patients

**Principal Investigator: Naomi Schlesinger, MD**

This consent form is part of an informed consent process for a research study and it will provide information that will help you to decide whether you wish to volunteer for this research study. It will help you to understand what the study is about and what will happen in the course of the Study.

If you have questions at any time during the research study, you should feel free to ask them and should expect to be given answers that you completely understand.

After all of your questions have been answered, if you still wish to take part in the study, you will be asked to sign this informed consent form.

You are not giving up any of your legal rights by volunteering for this research study or by signing this consent form. Your participation in this study is voluntary and you will not be penalized or lose benefits if you refuse to participate.

We invite you to participate in a clinical research study that is designed to help us explore whether exercise effects patients with gout.

There is no sponsor to this study. **We plan to recruit 250 subjects to this study.** Subjects will be adults between the ages of 18 and 89 who have been diagnosed with gout. Your participation in this study will last one visit. You will have one 30-minute visit to talk with your doctor and complete questionnaires after your scheduled appointment. We would appreciate if you could take several minutes to answer the attached questionnaires for our study examining whether there is an association between exercise and gout.

**Who is conducting this research study?**

Dr. Naomi Schlesinger is the Principal Investigator of this research study. A Principal Investigator has the overall responsibility for the conduct of the study. However, there are often other individuals who are part of the research team.

Dr. Schlesinger may be reached at phone number 732 235 8378

The study doctor Dr Schlesinger or another member of the study team (Dr. Alikhan Mariam) will also be asked to sign this informed consent. You will be given a copy of the signed consent form to keep.

**Why is this study being done?**

The goal of this study is to see if there is an association between exercise and gout.

**Why have you been asked to take part in this study?**

You have gout.

**Who may take part in this study? And who may not?**

Participants will be between the ages of 18 and 89 who have gout. You may not participate in this study if any of the following applies to you: 1) You are not between ages of 18 and 89, 2) You are not able to both read and speak English, 3) You have significant mental impairment, 4) You have a psychiatric disorder that would limit your ability to give informed consent or that might cause risk to you.

**How long will the study take and how many subjects will participate?**

You will be one of two hundred and fifty patients selected to participate. Your participation in this study requires approximately 30 minutes of your time today.

**What will you be asked to do if you take part in this research study?**

During the course of this study, the following will occur: You are to review this consent form, but not sign it until you have discussed any questions you might have with a study investigator. After reviewing and signing this consent form, you will complete four questionnaires.

**What are the risks and/or discomforts you might experience if you take part in this study?**

There are minimal risks to this study. All information provided by you will be kept strictly confidential.

**Are there any benefits for you if you choose to take part in this research study?**

The benefits of taking part in this study may be a sense of accomplishment in helping others to understand your disease better by being a research study participant. However, you may receive no direct benefit from taking part in this study.

**What are your alternatives if you don’t want to take part in this study?**

Your only alternative is not to take part in this study.

**Will there be any cost to you to take part in this study?**

There will be no cost for you to participate in this study.

**Will you be paid to take part in this study?**

You will not be paid for your participation in this research study.

**How will information about you be kept private or confidential?**

All efforts will be made to keep your personal information in your research record confidential, but total confidentiality cannot be guaranteed.

Your personal identity, that is your name, will be kept confidential. You will have a code number and your actual name will not be used. Only your study doctor will be able to link the code number to your name. Do not record your name so we can be sure to maintain the confidentiality of the responses.

**What will happen if you do not wish to take part in the study or if you later decide not to stay in the study?**

Participation in this study is voluntary. You may choose not to participate or you may change your mind at any time.

If you do not want to enter the study or decide to stop participating, your relationship with the study staff will not change, and you may do so without penalty and without loss of benefits to which you are otherwise entitled.

You may also withdraw your consent for the use of data already collected about you, but you must do this in writing to Dr Naomi Schlesinger, Department of Medicine, Rutgers RWJMS, MEB 468, New Brunswick, NJ 08903

**Who can you call if you have any questions?**

If you have any questions about taking part in this study .you can call the study doctor:

If you have any questions about taking part in this study, you can call the study doctor: Dr Schlesinger at 732 235 8378.

If you have any questions about your rights as a research subject, you can call the

IRB Director: (**732)-235-9806 and/or Human Subject Protection Program**
(732)-235-8578

**What are your rights if you decide to take part in this research study?**

You have the right to ask questions about any part of the study at any time. You should not sign this form unless you have had a chance to ask questions and have been given answers to all of your questions.

**PERMISSION (Authorization) TO USE OR SHARE HEALTH INFORMATION THAT IDENTIFIES YOU FOR A RESEARCH STUDY**

Information about you and your health is personal and private, so this information generally cannot be used in research without your written permission. The next few paragraphs tell you about how researchers want to use and share your health information in this research study. Your information will only be used as described here or as allowed or required by law. Ask questions if you do not understand any part of the research or the use of your health information. If you sign this consent form, you agree to let the researchers use your information in the research and share it with others as described below.

**What is the purpose of the research and how will my information be used?**

You are being invited to take part in this research study which is described at the beginning of this form. The purpose of collecting and using your health information for this study is to help researchers answer the questions that are being asked in the research.

**What information about me will be used?**

- Medical history or treatment
- Medications
- Laboratory/diagnostic tests or imaging

**Who may use, share or receive my information?**

The research team may use or share your information collected or created for this study with the following people and institutions:

- Rutgers University researchers involved in the study;
- The Rutgers University Institutional Review Board and Compliance Boards (a committee that reviews research studies to protect people participating in research).
- The Office for Human Research Protections in the U.S. Dept. of Health and Human Services (regulatory agency that oversees human subject research.

By taking part in this study, you should understand that the study collects demographic data and data on your health. This data will be recorded by the study doctor/investigator who may store and process your data with electronic data processing systems. The data will be kept as long as the study is being conducted and for six years*.*

Your personal identity, that is your name, will be kept confidential. You will have a code number and your actual name will not be used. Only your study doctor will be able to link the code number to your name and will keep this information for 6 years.

Your data may be used in scientific publications. If the findings from the study are published, you will not be identified by name. Your identity will be kept confidential. The exception to this rule will be when there is a court order or when a law exists requiring the study doctor to report communicable diseases. In this case, you will be informed of the intent to disclose this information to the state agency. Such a law exists in New Jersey for diseases such as cancer, infectious diseases such as hepatitis, HIV, viruses and many others.

The study doctor/investigator will be allowed to examine the data in order to analyze the information obtained from this study, and for general health research.

Those persons or organizations that receive your information may not be required by Federal privacy laws to protect it and may share your information with others without your permission, if permitted by the laws governing them.

**Will I be able to review my research record while the research is ongoing?**

No. We are not able to share information in the research records with you until the study is over. To ask for this information, please contact the Principal Investigator, the person in charge of this research study. If you have an interest in the aggregate results, they will be available once the study is over, please contact the Principal Investigator. **Do I have to give my permission?**

No. You do not have to permit use of your information. But, if you do not give permission, you cannot take part in this research study. (Saying no does not stop you from getting medical care or other benefits you are eligible for outside of this study.)

**If I say yes now, can I change my mind and take away my permission later?**

Yes. You may change your mind and not allow the continued use of your information (and to stop taking part in the study) at any time. If you take away permission, your information will no longer be used or shared in the study, but we will not be able to take back information that has already been used or shared with others. If you say yes now but change your mind later for use of your information in the research, you must write to the researcher and tell him or her of your decision: Dr Naomi Schlesinger, Department of Medicine, Rutgers RWJMS, MEB 468, New Brunswick, NJ 08903

**How long will my permission last?**

Your permission for the use and sharing of your health information will last until July 1, 2019

Thanking you in advance,

Naomi Schlesinger, MD

Professor of Medicine

Chief, Division of  Rheumatology

Department of Medicine
Rutgers – Robert Wood Johnson Medical School

MEB 468; PO Box 19
New Brunswick, NJ 08903-0019

Phone: 732 235 8378

**AGREEMENT TO PARTICIPATE**

**1. Subject consent:**

I have read this entire form, or it has been read to me, and I believe that I understand what has been discussed. All of my questions about this form or this study have been answered. I agree to take part in this research study.

Subject Name:

Subject Signature: Date:

**2. Signature of Investigator/Individual Obtaining Consent:**

To the best of my ability, I have explained and discussed the full contents of the study including all of the information contained in this consent form. All questions of the research subject have been accurately answered.

Investigator/Person Obtaining Consent (printed name):

Signature: Date:
